# 3-(1-Phenyl­ethyl)-1,3-thia­zinane-2-thione

**DOI:** 10.1107/S1600536809046248

**Published:** 2009-11-11

**Authors:** Fu-feng Yan, Chong-jia Liang

**Affiliations:** a Provincial Key Laboratory of Surface & Interface Science, Zhengzhou University of Light Industry, Zhengzhou 450002, People’s Republic of China; b Henan Sports School, Zhengzhou 450044, People’s Republic of China

## Abstract

In the title mol­ecule, C_12_H_15_NS_2_, the 1,3-thia­zinane ring has a half-boat conformation; the C atom at position 5 deviates by 0.715 (2) Å from the mean plane (*P*) of the remaining five atoms. Plane *P* and the phenyl ring form a dihedral angle of 83.62 (3)°. In the crystal structure, weak inter­molecular C—H⋯S hydrogen bonds link mol­ecules related by translation along the axis *a* into chains.

## Related literature

For the crystal structures of related thia­zinane derivatives, see: Kálmán *et al.* (1977 ▶); Peng & Wu (2009 ▶); Amir *et al.* (2006 ▶). For the biological activity of thia­zinane-containing compounds, see: Soloway *et al.* (1978 ▶); Tomizawa *et al.* (1995 ▶).
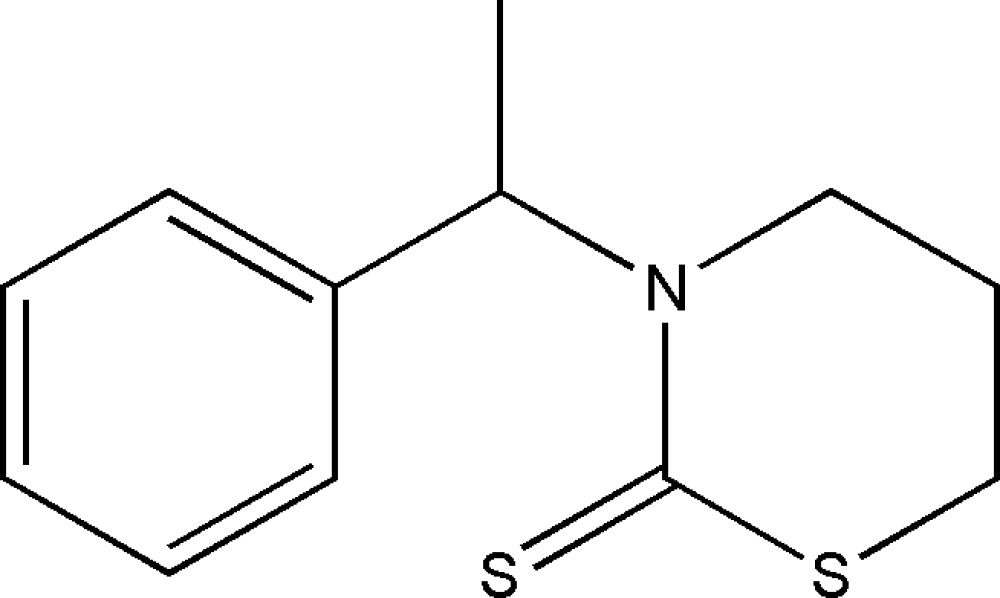


## Experimental

### 

#### Crystal data

C_12_H_15_NS_2_
*M* *_r_* = 237.37
Monoclinic, 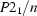

*a* = 7.0169 (4) Å
*b* = 15.5107 (9) Å
*c* = 11.0349 (7) Å
β = 102.391 (3)°
*V* = 1173.03 (12) Å^3^
*Z* = 4
Mo *K*α radiation
μ = 0.42 mm^−1^
*T* = 113 K
0.26 × 0.10 × 0.08 mm

#### Data collection

Rigaku Saturn diffractometer
Absorption correction: multi-scan (*CrystalClear*; Rigaku, 2005 ▶) *T*_min_ = 0.899, *T*_max_ = 0.967
14476 measured reflections
2798 independent reflections
2631 reflections with *I* > 2σ(*I*)
*R*_int_ = 0.040

#### Refinement

*R*[*F* ^2^ > 2σ(*F* ^2^)] = 0.041
*wR*(*F* ^2^) = 0.087
*S* = 1.13
2798 reflections
137 parameters
H-atom parameters constrained
Δρ_max_ = 0.27 e Å^−3^
Δρ_min_ = −0.32 e Å^−3^

### 

Data collection: *CrystalClear* (Rigaku, 2005 ▶); cell refinement: *CrystalClear*; data reduction: *CrystalClear*; program(s) used to solve structure: *SHELXTL* (Sheldrick, 2008 ▶); program(s) used to refine structure: *SHELXTL*; molecular graphics: *SHELXTL*; software used to prepare material for publication: *SHELXTL* .

## Supplementary Material

Crystal structure: contains datablocks I, global. DOI: 10.1107/S1600536809046248/cv2647sup1.cif

Structure factors: contains datablocks I. DOI: 10.1107/S1600536809046248/cv2647Isup2.hkl

Additional supplementary materials:  crystallographic information; 3D view; checkCIF report

## Figures and Tables

**Table 1:** Hydrogen-bond geometry (Å, °)

*D*—H⋯*A*	*D*—H	H⋯*A*	*D*⋯*A*	*D*—H⋯*A*
C6—H6*C*⋯S1^i^	0.98	2.76	3.7279 (17)	168

Symmetry code: (i) 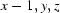.

**Fig. 1.**
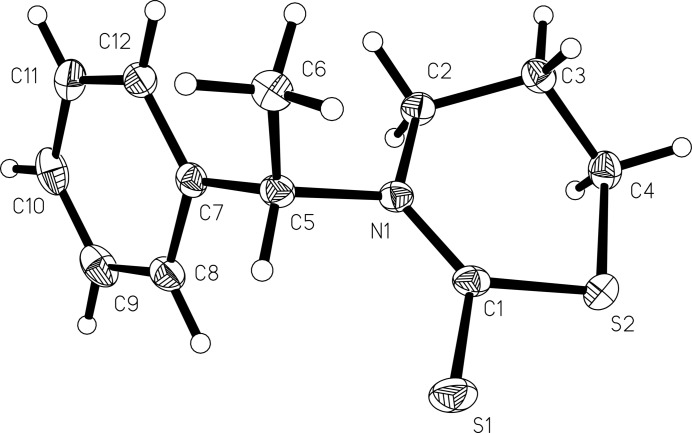
The molecular structure of (I) with displacement ellipsoids drawn at the 40% probability level.

## supplementary crystallographic information

### Comment

Many compounds containing thiazinane groups possess a broad spectrum of biological activities (Soloway *et al.*, 1978; Tomizawa *et al.*, 1995). Herein we report the crystal structure of the title compound, (I).

In (I) (Fig. 1), all bond lengths and angles are in a good agreement with those reported previously (Kálmán *et al.*, 1977; Peng & Wu, 2009; Amir *et al.*, 2006). The thiazinane ring shows a conformation near to a half boat with the carbon atom at position 5 (C3) deviating 0.715 (2) Å above the plane p1 formed by S2, N1, C1, C2 and C4 [maximum least squares plane deviation for S2 0.038 (3) Å]. The dihedral angle between the benzene ring C7-C12 and plane p1 is 83.62 (3) °. In the crystal structure, weak intermolecular C—H···S hydrogen bonds link molecules related by translation along axis *a* into chains.

### Experimental

A solution of 1,3-thiazinane-2-thione (1.33 g, 10 mmol) and sodium hydride (0.3 g) dissolved in anhydrous acetonitrile (20 ml), and dropwise added over a period of 10 min to a solution of 1-(1-chloroethyl)benzene (1.41g, 10 mmol) in acetonitrile (10 ml) at 273 K. The mixture was stirred at 353 K for 2 h. The solvent was removed and the residue was purified by flash chromatography (3:1 Cyclohexane:Dichloromethane) to give title compound as a white solid (1.90 g, 80%). Single crystals suitable for X-ray measurements were obtained by recrystallization from ethanol at room temperature.

### Refinement

C-bound H atoms were placed in calculated positions (C—H = 0.95–1.00 Å), and included in the final cycles of refinement using a riding model, with *U*_iso_(H) = 1.2*U*_eq_(C) for the aryl and methylene H atoms and 1.5*U*_eq_(C) for the methyl H atoms.

### Crystal data

**Table:**

C_12_H_15_NS_2_	*F*(000) = 504
*M**_r_* = 237.37	*D*_x_ = 1.344 Mg m^−^^3^
Monoclinic, *P*2_1_/*n*	Mo *K*α radiation, λ = 0.71070 Å
Hall symbol: -P 2yn	Cell parameters from 3792 reflections
*a* = 7.0169 (4) Å	θ = 2.3–27.9°
*b* = 15.5107 (9) Å	µ = 0.42 mm^−^^1^
*c* = 11.0349 (7) Å	*T* = 113 K
β = 102.391 (3)°	Prism, colourless
*V* = 1173.03 (12) Å^3^	0.26 × 0.10 × 0.08 mm
*Z* = 4	

### Data collection

**Table:**

Rigaku Saturn diffractometer	2798 independent reflections
Radiation source: rotating anode	2631 reflections with *I* > 2σ(*I*)
confocal	*R*_int_ = 0.040
Detector resolution: 14.63 pixels mm^-1^	θ_max_ = 27.9°, θ_min_ = 2.3°
ω scans	*h* = −9→9
Absorption correction: multi-scan (*CrystalClear*; Rigaku, 2005)	*k* = −19→20
*T*_min_ = 0.899, *T*_max_ = 0.967	*l* = −14→14
14476 measured reflections	

### Refinement

**Table:**

Refinement on *F*^2^	Primary atom site location: structure-invariant direct methods
Least-squares matrix: full	Secondary atom site location: difference Fourier map
*R*[*F*^2^ > 2σ(*F*^2^)] = 0.041	Hydrogen site location: inferred from neighbouring sites
*wR*(*F*^2^) = 0.087	H-atom parameters constrained
*S* = 1.13	*w* = 1/[σ^2^(*F*_o_^2^) + (0.034*P*)^2^ + 0.4071*P*] where *P* = (*F*_o_^2^ + 2*F*_c_^2^)/3
2798 reflections	(Δ/σ)_max_ < 0.001
137 parameters	Δρ_max_ = 0.27 e Å^−^^3^
0 restraints	Δρ_min_ = −0.32 e Å^−^^3^

### Special details

**Table:**

Geometry. All e.s.d.'s (except the e.s.d. in the dihedral angle between two l.s. planes) are estimated using the full covariance matrix. The cell e.s.d.'s are taken into account individually in the estimation of e.s.d.'s in distances, angles and torsion angles; correlations between e.s.d.'s in cell parameters are only used when they are defined by crystal symmetry. An approximate (isotropic) treatment of cell e.s.d.'s is used for estimating e.s.d.'s involving l.s. planes.
Refinement. Refinement of *F*^2^ against ALL reflections. The weighted *R*-factor *wR* and goodness of fit *S* are based on *F*^2^, conventional *R*-factors *R* are based on *F*, with *F* set to zero for negative *F*^2^. The threshold expression of *F*^2^ > σ(*F*^2^) is used only for calculating *R*-factors(gt) *etc*. and is not relevant to the choice of reflections for refinement. *R*-factors based on *F*^2^ are statistically about twice as large as those based on *F*, and *R*- factors based on ALL data will be even larger.

### Fractional atomic coordinates and isotropic or equivalent isotropic displacement parameters (Å^2^)

**Table:**

	*x*	*y*	*z*	*U*_iso_*/*U*_eq_	
S1	1.23060 (6)	0.17662 (3)	0.70183 (4)	0.03009 (13)	
S2	1.02102 (6)	0.13716 (3)	0.45696 (4)	0.02633 (12)	
N1	0.86777 (18)	0.11187 (8)	0.65929 (12)	0.0193 (3)	
C1	1.0203 (2)	0.13813 (10)	0.61536 (15)	0.0208 (3)	
C2	0.6824 (2)	0.08125 (10)	0.58027 (14)	0.0215 (3)	
H2A	0.5755	0.0905	0.6247	0.026*	
H2B	0.6923	0.0185	0.5663	0.026*	
C3	0.6315 (2)	0.12648 (10)	0.45586 (15)	0.0225 (3)	
H3A	0.4993	0.1088	0.4115	0.027*	
H3B	0.6302	0.1896	0.4690	0.027*	
C4	0.7771 (2)	0.10468 (11)	0.37795 (15)	0.0262 (4)	
H4A	0.7410	0.1346	0.2970	0.031*	
H4B	0.7749	0.0418	0.3621	0.031*	
C5	0.8766 (2)	0.11054 (10)	0.79582 (14)	0.0207 (3)	
H5	1.0167	0.1176	0.8383	0.025*	
C6	0.7662 (2)	0.18756 (10)	0.83194 (15)	0.0245 (3)	
H6A	0.8277	0.2410	0.8124	0.037*	
H6B	0.7692	0.1854	0.9211	0.037*	
H6C	0.6305	0.1858	0.7855	0.037*	
C7	0.8115 (2)	0.02299 (10)	0.83270 (14)	0.0203 (3)	
C8	0.9420 (3)	−0.04584 (11)	0.84269 (15)	0.0250 (4)	
H8	1.0682	−0.0366	0.8268	0.030*	
C9	0.8901 (3)	−0.12754 (11)	0.87548 (16)	0.0316 (4)	
H9	0.9796	−0.1740	0.8804	0.038*	
C10	0.7074 (3)	−0.14124 (11)	0.90105 (16)	0.0316 (4)	
H10	0.6718	−0.1970	0.9243	0.038*	
C11	0.5777 (3)	−0.07356 (11)	0.89258 (15)	0.0283 (4)	
H11	0.4528	−0.0828	0.9105	0.034*	
C12	0.6284 (2)	0.00820 (10)	0.85792 (14)	0.0235 (3)	
H12	0.5373	0.0542	0.8515	0.028*	

### Atomic displacement parameters (Å^2^)

**Table:**

	*U*^11^	*U*^22^	*U*^33^	*U*^12^	*U*^13^	*U*^23^
S1	0.0166 (2)	0.0355 (3)	0.0367 (3)	−0.00502 (17)	0.00244 (18)	−0.00113 (19)
S2	0.0240 (2)	0.0303 (2)	0.0266 (2)	−0.00098 (17)	0.00950 (17)	0.00309 (17)
N1	0.0157 (6)	0.0211 (6)	0.0203 (6)	−0.0016 (5)	0.0020 (5)	−0.0005 (5)
C1	0.0177 (7)	0.0165 (7)	0.0282 (8)	0.0027 (6)	0.0048 (6)	0.0007 (6)
C2	0.0181 (7)	0.0238 (8)	0.0217 (8)	−0.0045 (6)	0.0022 (6)	−0.0013 (6)
C3	0.0214 (8)	0.0218 (8)	0.0225 (8)	−0.0018 (6)	0.0009 (6)	0.0000 (6)
C4	0.0290 (9)	0.0280 (9)	0.0211 (8)	−0.0021 (7)	0.0043 (7)	0.0007 (7)
C5	0.0178 (7)	0.0244 (8)	0.0188 (7)	0.0005 (6)	0.0015 (6)	−0.0017 (6)
C6	0.0259 (8)	0.0218 (8)	0.0245 (8)	0.0006 (7)	0.0025 (7)	−0.0032 (6)
C7	0.0224 (8)	0.0226 (8)	0.0149 (7)	0.0008 (6)	0.0016 (6)	−0.0019 (6)
C8	0.0257 (8)	0.0275 (8)	0.0204 (8)	0.0067 (7)	0.0019 (6)	−0.0023 (6)
C9	0.0421 (11)	0.0241 (8)	0.0249 (8)	0.0105 (8)	−0.0008 (8)	−0.0027 (7)
C10	0.0484 (11)	0.0207 (8)	0.0227 (8)	−0.0016 (8)	0.0012 (8)	0.0016 (7)
C11	0.0328 (9)	0.0301 (9)	0.0225 (8)	−0.0051 (7)	0.0071 (7)	0.0019 (7)
C12	0.0251 (8)	0.0241 (8)	0.0210 (8)	0.0033 (7)	0.0043 (6)	0.0002 (6)

### Geometric parameters (Å, °)

**Table:**

S1—C1	1.6851 (16)	C5—H5	1.0000
S2—C1	1.7491 (17)	C6—H6A	0.9800
S2—C4	1.8172 (17)	C6—H6B	0.9800
N1—C1	1.330 (2)	C6—H6C	0.9800
N1—C2	1.4803 (19)	C7—C12	1.390 (2)
N1—C5	1.495 (2)	C7—C8	1.395 (2)
C2—C3	1.515 (2)	C8—C9	1.388 (2)
C2—H2A	0.9900	C8—H8	0.9500
C2—H2B	0.9900	C9—C10	1.387 (3)
C3—C4	1.508 (2)	C9—H9	0.9500
C3—H3A	0.9900	C10—C11	1.379 (3)
C3—H3B	0.9900	C10—H10	0.9500
C4—H4A	0.9900	C11—C12	1.393 (2)
C4—H4B	0.9900	C11—H11	0.9500
C5—C7	1.516 (2)	C12—H12	0.9500
C5—C6	1.523 (2)		
			
C1—S2—C4	106.11 (8)	N1—C5—H5	107.2
C1—N1—C2	123.90 (13)	C7—C5—H5	107.2
C1—N1—C5	120.59 (13)	C6—C5—H5	107.2
C2—N1—C5	115.50 (12)	C5—C6—H6A	109.5
N1—C1—S1	125.27 (13)	C5—C6—H6B	109.5
N1—C1—S2	122.45 (12)	H6A—C6—H6B	109.5
S1—C1—S2	112.27 (9)	C5—C6—H6C	109.5
N1—C2—C3	113.06 (13)	H6A—C6—H6C	109.5
N1—C2—H2A	109.0	H6B—C6—H6C	109.5
C3—C2—H2A	109.0	C12—C7—C8	118.47 (15)
N1—C2—H2B	109.0	C12—C7—C5	123.11 (14)
C3—C2—H2B	109.0	C8—C7—C5	118.42 (14)
H2A—C2—H2B	107.8	C9—C8—C7	120.99 (16)
C4—C3—C2	110.86 (13)	C9—C8—H8	119.5
C4—C3—H3A	109.5	C7—C8—H8	119.5
C2—C3—H3A	109.5	C10—C9—C8	119.90 (16)
C4—C3—H3B	109.5	C10—C9—H9	120.0
C2—C3—H3B	109.5	C8—C9—H9	120.0
H3A—C3—H3B	108.1	C11—C10—C9	119.66 (16)
C3—C4—S2	110.36 (11)	C11—C10—H10	120.2
C3—C4—H4A	109.6	C9—C10—H10	120.2
S2—C4—H4A	109.6	C10—C11—C12	120.52 (17)
C3—C4—H4B	109.6	C10—C11—H11	119.7
S2—C4—H4B	109.6	C12—C11—H11	119.7
H4A—C4—H4B	108.1	C7—C12—C11	120.44 (15)
N1—C5—C7	109.47 (12)	C7—C12—H12	119.8
N1—C5—C6	109.85 (13)	C11—C12—H12	119.8
C7—C5—C6	115.64 (13)		
			
C2—N1—C1—S1	177.75 (11)	C2—N1—C5—C6	−78.27 (16)
C5—N1—C1—S1	−3.2 (2)	N1—C5—C7—C12	−103.30 (16)
C2—N1—C1—S2	−1.5 (2)	C6—C5—C7—C12	21.4 (2)
C5—N1—C1—S2	177.55 (11)	N1—C5—C7—C8	77.19 (17)
C4—S2—C1—N1	4.98 (15)	C6—C5—C7—C8	−158.12 (14)
C4—S2—C1—S1	−174.33 (8)	C12—C7—C8—C9	0.8 (2)
C1—N1—C2—C3	−33.2 (2)	C5—C7—C8—C9	−179.67 (15)
C5—N1—C2—C3	147.72 (13)	C7—C8—C9—C10	−1.2 (3)
N1—C2—C3—C4	66.05 (17)	C8—C9—C10—C11	0.6 (3)
C2—C3—C4—S2	−59.48 (16)	C9—C10—C11—C12	0.4 (3)
C1—S2—C4—C3	25.10 (14)	C8—C7—C12—C11	0.2 (2)
C1—N1—C5—C7	−129.39 (14)	C5—C7—C12—C11	−179.36 (15)
C2—N1—C5—C7	49.72 (17)	C10—C11—C12—C7	−0.7 (2)
C1—N1—C5—C6	102.62 (16)		

### Hydrogen-bond geometry (Å, °)

**Table:**

*D*—H···*A*	*D*—H	H···*A*	*D*···*A*	*D*—H···*A*
C6—H6C···S1^i^	0.98	2.76	3.7279 (17)	168

Symmetry codes: (i) *x*−1, *y*, *z*.
